# Assessment of human papillomavirus E6/E7 oncogene expression as cervical disease biomarker

**DOI:** 10.1186/s12885-016-2885-x

**Published:** 2016-11-05

**Authors:** Nerea Fontecha, Miren Basaras, Silvia Hernáez, Daniel Andía, Ramón Cisterna

**Affiliations:** 1 Department of Immunology, Microbiology and Parasitology, Medicine and Odontology Faculty, University of Basque Country (UPV/EHU), Sarriena auzoa, 48940 Leioa-Bizkaia, Spain; 2 Clinical Microbiology and Infection Control Department, Basurto University Hospital, Bilbao, 48013 Spain; 3 Department of Obstetrics and Gynaecology, Basurto University Hospital, Bilbao, 48013 Spain

**Keywords:** High-risk HPV genotypes, E6/E7 mRNA detection, Oncogenes, Biomarker, Prognosis marker

## Abstract

**Background:**

The aims of this study were to detect HPV E6/E7 mRNA expression in women with high-risk genotypes (HPV-16, -18, -31, -33 and -45) analysing its relationship with tissue pathology and 2) 2-year follow-up of E6/E7 mRNA tested group.

**Methods:**

Our samples were genotyped and classified by pathologists according to Bethesda system. After RNA extraction, E6/E7 oncogene mRNA detection was performed by NucliSens® EasyQ® HPV v1 Test (bioMérieux).

**Results:**

The results of the present study showed that E6/E7 mRNA positivity rate was 68.29 % in women tested once and 69.56 % in women tested twice. According to tissue pathology, all samples with high-grade lesions were positive for mRNA. Among women with low-grade lesions varied over the years from 89.28 to 84 % in women tested once and from 77.77 to 70 % in tested twice. Among women without lesion, positivity rate maintained in women tested once (from 50 to 41.38 %) and decreased in tested twice, from 63.63 to 44.44 %. Regarding lesion evolution, mRNA positivity was higher in women with lesion progression (53.13 %) and in women with positive results in two tested samples (83.33 %).

**Conclusion:**

HPV E6/E7 mRNA detection may be an effective screening test and biomarker for cervical cancer in women infected with these five genotypes. Nonetheless, further studies are needed to standardize as routine triage test.

## Background

Human papillomavirus (HPV) infection is responsible of cervical cancer, which is the second most frequent cancer affecting woman worldwide [1–3].

More than 100 genotypes have been described; 12 of them are classified as high-risk genotypes (HR-HPV) due to their high oncogenic potential [4]. Among these HR-HPV, 16 and 18 genotypes are responsible for 76 % of cervical cancer in Europe [5]. The most frequent in Spain in overall women population are 16, 52, 51, 31 and 66 [6] whereas in our area, northern Spain, the most common genotypes in women with abnormal cytology are 16, 51, 53, 52, 39, 18, 58 and 66 [7]. On the other hand, HPV-16, -18, -33 and -45 are more often found in invasive cervical carcinoma rather than precancerous lesions [8]. Moreover, HPV-16 and HPV-33 increase the risk of developing a grade 3 cervical intraepithelial neoplasia [8, 9].

HPV viral oncoprotein expression differs among infection state (active, latent, or persistent) [10, 11]. HPV E6 and E7 oncogene deregulation has been shown as a crucial factor in neoplasic lesions progression [12]. Moreover, E6 and E7 oncoprotein continuous expression is essential to maintain the neoplastic growth features [13].

To date, cervical cancer screening is based on cytology and this has been a powerful tool to reduce the incidence and mortality of this type of cancer [14, 15], but this strategy is not completely effective to predict invasive cervical cancer [16]. Recently, DNA detection is also used [17]. Nevertheless, DNA detection gives information about the virus presence but not about the infection state. Thus, it is crucial to find new biomarkers with high positive predictive value which can be used in cervical cancer screening. E6/E7 oncogene mRNA expression could be the most promising cervical cancer biomarker according to current data [18]. Moreover, it has been observed that mRNA may be more adequate than cytology for HPV infected women follow-up [19, 20] owing to its prognostic value.

The present study was designed to 1) study HPV E6/E7 mRNA expression and analyse its relationship with tissue pathology in order to test mRNA as an effective biomarker for cervical cancer and 2) 2-year follow-up of a group of women and test E6/E7 mRNA expression.

## Methods

### Samples

Samples from women which were referred from Consultation of Sexually Transmitted Diseases and Gynaecological consultation at Basurto University Hospital (Basque Country, Spain) were analyzed due to possible HPV infection during the last 7 years (2007–2014). All patients gave written and informed consent prior to their inclusion in the study.

The samples were categorized by pathologists according to Bethesda system: 1) negative (no lesion was found) 2) Atypical squamous cells of undetermined significance (ASCUS) 3) Low-grade squamous intraepithelial lesion (LSIL) and 4) High-grade squamous intraepithelial lesion (HSIL).

### Molecular genotyping

Samples were analyzed with Cobas® HPV Test (Roche Molecular Diagnostics, Mannheim, Germany) that detects 14 high-risk HPV DNA genotypes, 16 and 18 separately and other 12 high-risk HPV genotypes altogether. Samples that were positive for other HR were genotyped with Linear Array HPV Genotyping Test kit (Roche Molecular Diagnostics), a line-blot assay that detects 37 genotypes (6, 11, 16, 18, 26, 31, 33, 35, 39, 40, 42, 45, 51, 52, 53, 54, 55, 56, 58, 59, 61, 62, 64, 66, 67, 68, 69, 70, 71, 72, 73, 81, 82, 83, 84, IS39, and CP6108).

### RNA extraction

RNA was extracted from 200 μl of samples by NucliSENS Lysis Buffer and NucliSENS® miniMAG® (bioMérieux, Marcy l’Etoile, France) according to manufacturer’s instructions.

### E6/E7 mRNA study

E6/E7 oncogene mRNA was detected by NucliSens® EasyQ® HPV v1 Test (bioMérieux) following manufacturer’s instructions. This test detects the E6/E7 oncogene expression of five HR-HPV genotypes (16, 18, 31, 33 and 45). The quality of extracted RNA was monitored by an internal control: human U1 small nuclear ribonucleoprotein specific protein A (U1A). If neither U1A nor mRNA were detected, the test result was analyzed as invalid. Invalid runs were repeated once again.

## Results

In the present study, E6/E7 mRNA expression was analyzed in a total of 128 samples (corresponding to 105 women). Eighty-two women’s samples were analyzed once and in 23 women mRNA expression was studied twice (in 2012/2013 and 2014, a total of 46 samples). All these women were positive for HPV DNA (DNA positivity rate was 100 %).

HPV-16 was the most prevalent genotype (68.71 %, 101/147), followed by 18 (12.25 %, 18/147), 45 (8.16 %, 12/147), 31 (6.80 %, 10/147) and 33 (4.08 %, 6/147). Nevertheless, regarding oncogene expression, HPV-33 was the most expressed (100 %, 6/6), followed by 18 (77.77 %, 14/18), 16 (70.29 %, 71/101), 31 (70 %, 7/10) and 45 (25 %, 3/12).

### mRNA expression in women tested once

Eighty-two women samples were tested for E6/E7 mRNA oncogene expression only once. The mean age was 33.62 ± 10.57 years (range 17–63). Most of them were infected with multiple HPV infection genotypes (54/82) whereas 34.15 % of them had single HPV genotype infection.

More frequently detected genotype was 16 (67.01 %) followed by 18, 45, 31 and 33 (11.34, 10.31, 7.21 and 4.13 %, respectively).

The E6/E7 mRNA positivity rate was 68.29 % (56/82). The mean age of E6/E7 positive and negative women was similar (33.53 ± 11.12 vs 33.8 ± 9.62 years). HPV 16 E6/E7 mRNA was detected in 71.42 % of positive samples (40/56), 18 in 10.71 % (6/56), 33 in 3.57 % (2/56), 16 plus 33 multiple infection in 3.57 % (2/56), and the following genotypes were only detected in 1.78 % of positive samples (1/56) (31; 45; 16, 18, 31 plus 45; 16, 18 plus 33; 16, 31 plus 45 and 16 plus 31 genotype).

**Fig. 1:**
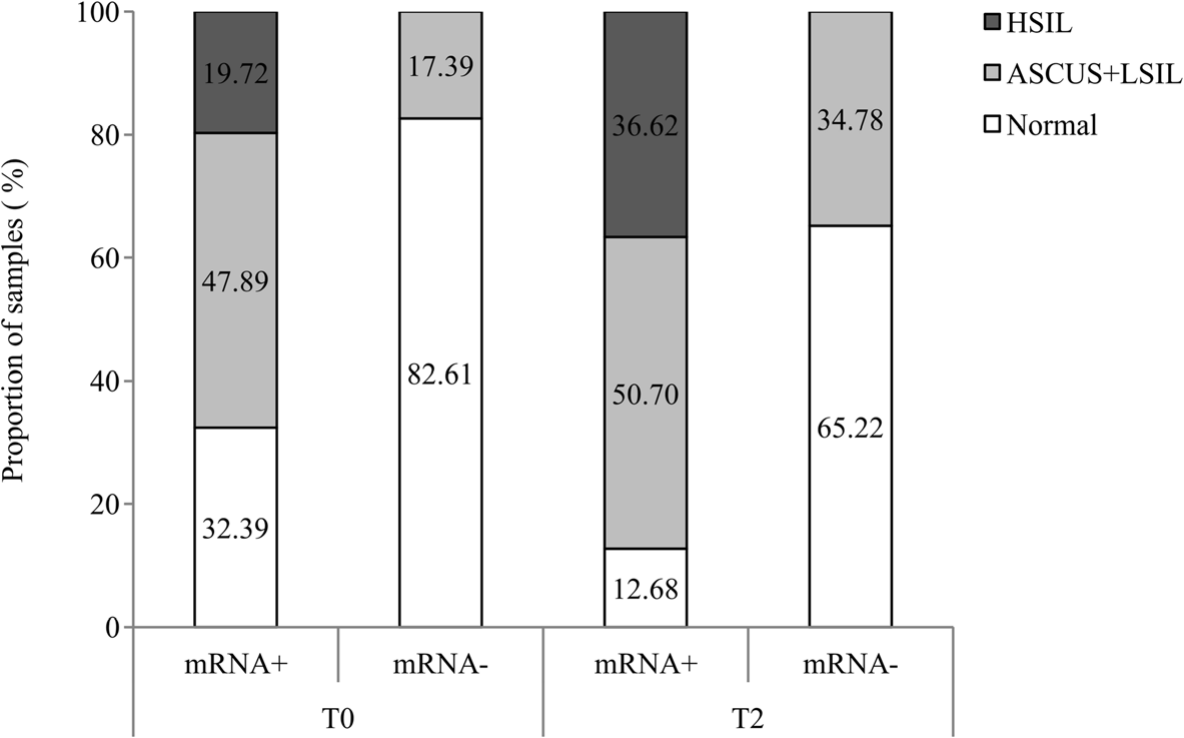
Women tissue pathology conforming to E6/E7 mRNA positivity and negativity rates in women tested once. E6/E7 mRNA rates: positivity rate for E6/E7 mRNA expression (mRNA+) and negativity rate for E6/E7 mRNA expression (mRNA-). T0 corresponds to the time when the sample was collected and T2 corresponds to next 2 years cytology results (without E6/E7 mRNA expression test). Tissue pathology was divided into three groups: 1) normal (no lesion), 2) Atypical squamous cells of undetermined significance (ASCUS) and low-grade squamous intraepithelial lesion (LSIL) and 3) High-grade squamous intraepithelial lesion (HSIL)

The E6/E7 mRNA results were analyzed regarding their cytology status. There were 77 women with cytology and E6/E7 mRNA test. Pathogenicity was divided into three groups: 1) normal or negative (no lesion was found), 2) ASCUS and LSIL and 3) HSIL. Among E6/E7 mRNA positive samples, 47.89 % belonged to women with ASCUS or LSIL (Fig. 1). The mRNA negative rate for normal samples was 82.61 and 17.39 % for ASCUS or LSIL women specimens. On the other hand, 77 women clinical follow-up was made in the following years based on cytological results. Taking into account samples pathogenicity, among E6/E7 mRNA positive samples, 50.70 % belong to women with ASCUS or LSIL and 36.62 % with HSIL. In addition, among women without E6/E7 mRNA expression, 65.22 % presented normal pathology results.

Furthermore, E6/E7 mRNA was studied according to each pathology groups (Table 1). Regarding women follow-up (two years later) among women that had HSIL lesions E6/E7 mRNA positivity rate was 100 % (remains constant throughout the years) and among ASCUS and LSIL specimens varied from 89.28 to 82.60 %. In addition, E6/E7 mRNA negativity rate increased from 51.28 to 59.38 %.

**Table 1:** E6/E7 mRNA detection according to cytology results

			E6/E7mRNA expression (n/%)	
		Cytology result	mRNA positive	mRNA negative
mRNA once tested samples (*N* = 77)	T0	Normal	19/48.72	20/51.28
		ASCUS + LSIL	25/89.28	3/10.72
		HSIL	10/100.00	0/0
	T2	Normal	13/40.62	19/59.38
		ASCUS + LSIL	19/82.60	4/17.40
		HSIL	22/100.00	0/0.00
mRNA twice tested samples (*N* = 23)	T0	Normal	7/63.63	4/36.37
		ASCUS + LSIL	7/77.77	2/22.23
		HSIL	3/100.00	0/0.00
	T2	Normal	4/44.44	5/55.56
		ASCUS + LSIL	7/70.00	3/30.00
		HSIL	4/100.00	0/0.00

T0 corresponds to the time when the sample was collected and mRNA was analysed and T2 corresponds to next 2 years pathology progressions based on cytological results. Cytology results were divided into three groups: 1) normal (no lesion), 2) Atypical squamous cells of undetermined significance (ASCUS) and low-grade squamous intraepithelial lesion (LSIL) and 3) High-grade squamous intraepithelial lesion (HSIL)

**Fig. 2:**
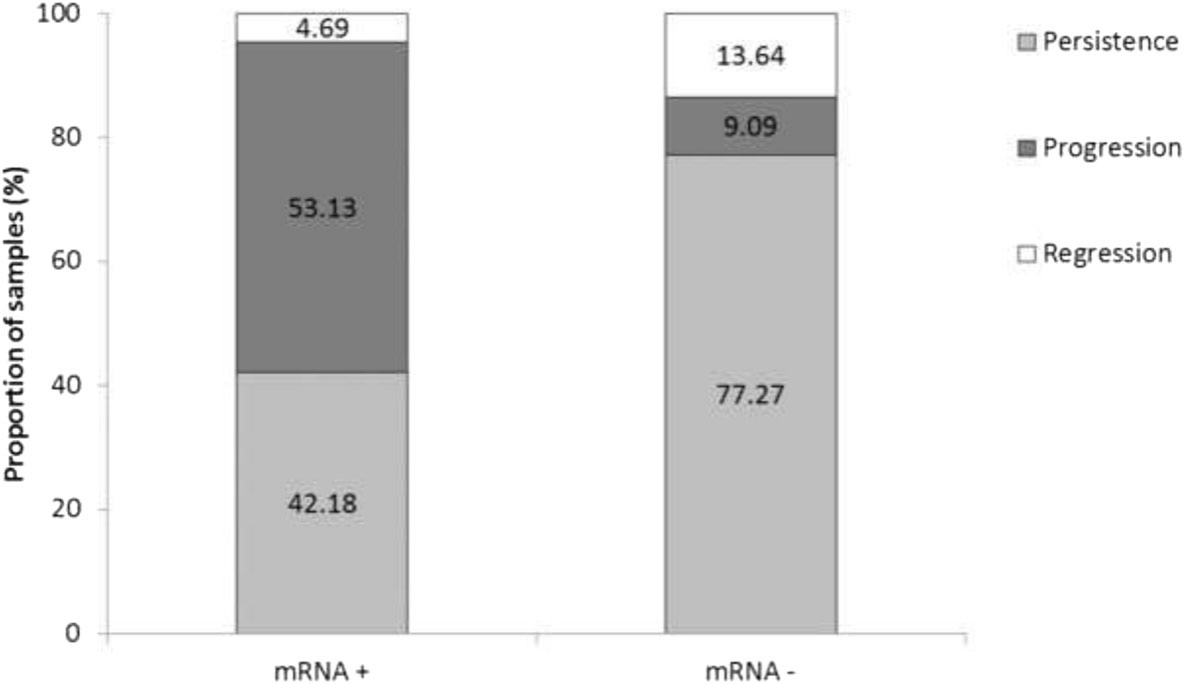
Lesion progression and its relationship with E6/E7 mRNA expression in women tested once. E6/E7 mRNA rates: positivity rate for E6/E7 mRNA expression (mRNA+) and negativity rate for E6/E7 mRNA expression (mRNA-). Lesion progression was categorized into three groups: 1) persistence: samples with the same grade of lesion, 2) progression: specimens with worsen lesion and 3) clearance: women who had had a lesion clearance during the next years after sample collection

On the other hand, lesion progression was assessed observing cytology results of those women in the following years (Fig. 2). Samples were categorized into three groups: 1) persistence: samples with the same grade of lesion (there was not a worsen process or clearance), 2) progression: specimens with worsened lesion (the lesion had worsened during the next years after sample collection) and 3) regression: women who had had a lesion clearance. The mRNA positivity rate was the highest in women who had suffered from lesion worsening process (53.13 %) and lower in women who had had the same lesion (42.18 %) and who had cleared the lesion over time (4.69 %). Moreover, there was a high correlation between mRNA negativity rate and samples with the same grade of lesion and clearance specimens (77.27 and 13.64 %, respectively).

### mRNA expression in women tested twice

Twenty-three women were tested twice in samples collected in two different years. The mean age was 38.87 ± 11.93 (range 24–58 years). HPV single infection was detected in 52.17 % (12/23) and multiple HPV infection in 47.83 % (11/23). The most detected genotype was 16 (73.47 %) followed by 18 (14.29 %) and 31, 33 and 45 genotypes (4.08 % of incidence each one).

The E6/E7 mRNA positivity rate was 69.56 % (32/46) and the oncogene expression was mostly detected in HPV-16 in 68.75 % of samples (22/32), followed by 18 in 15.63 % (5/32), 16 plus 31 multiple infection in 6.26 % (2/32), and the followings genotypes in 3.12 % (1/32): 33; 16 plus 18 and 16, 31 plus 33.

**Fig. 3:**
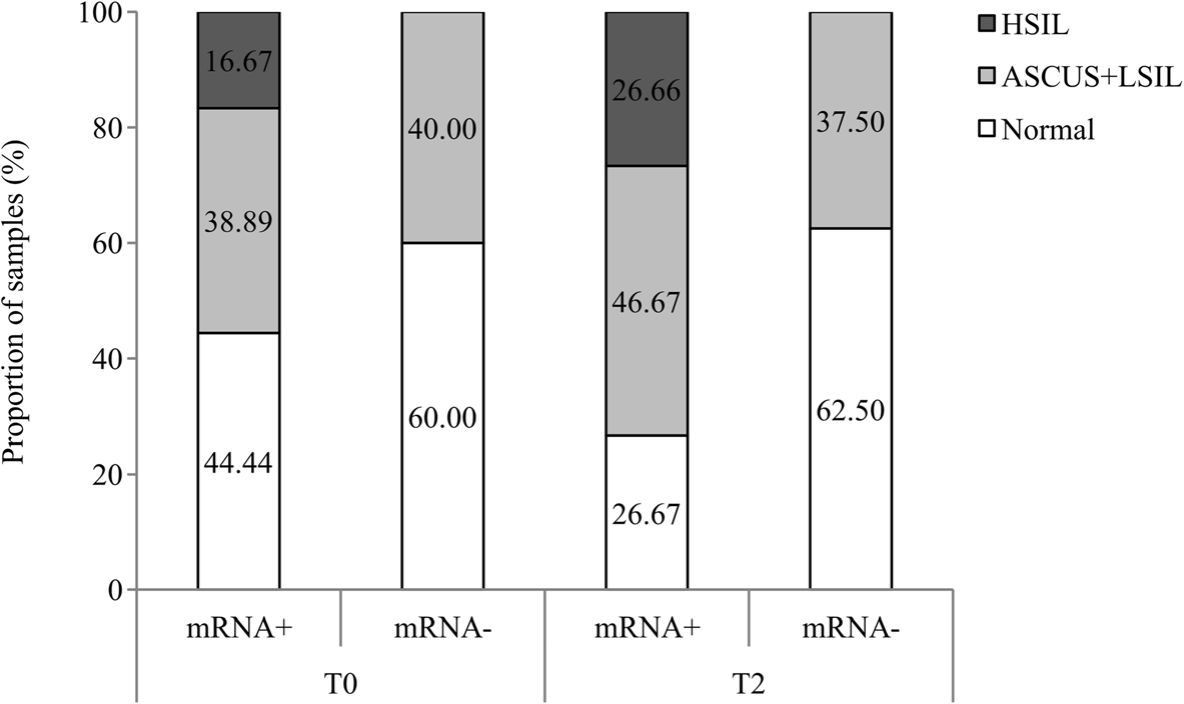
Patients tissue pathology according to E6/E7 mRNA positivity and negativity rates in women that were tested twice. E6/E7 mRNA rates: positivity rate for E6/E7 mRNA expression (mRNA+) and negativity rate for E6/E7 mRNA expression (mRNA-). T0 corresponds to results from the first time collected sample and T2 corresponds to next years collected specimen results. Tissue pathology was classified into three groups: 1) normal (no lesion), 2) Atypical squamous cells of undetermined significance (ASCUS) and low-grade squamous intraepithelial lesion (LSIL) and 3) High-grade squamous intraepithelial lesion (HSIL)

According to E6/E7 mRNA positivity rate, 55.56 % had some type of lesion (38.89 % ASCUS or LSIL and 16.67 % HSIL) in samples taken first (Fig. 3). However, the percentage of women with some type of lesion and mRNA positivity increased substantially in samples taken during the following years (46.67 % in women with ASCUS or LSIL and 26.66 % with HSIL). On the other hand, E6/E7 mRNA negativity rate remained stable over time in women with ASCUS or LSIL (from 40 to 37.5 %) and in women without lesion (from 60 to 62.5 %).

Otherwise, concerning pathology groups, among women with ASCUS or LSIL and HSIL mRNA rate remained stable (from 77.77 to 70 % and 100 %) (Table 1). Among women without lesion, the positivity rate decreased over the years (from 63.63 to 44.44 %). Regarding E6/E7 mRNA negativity rate, it was observed an increase among samples without lesion (from 36.37 to 55.56 %).

**Fig. 4:**
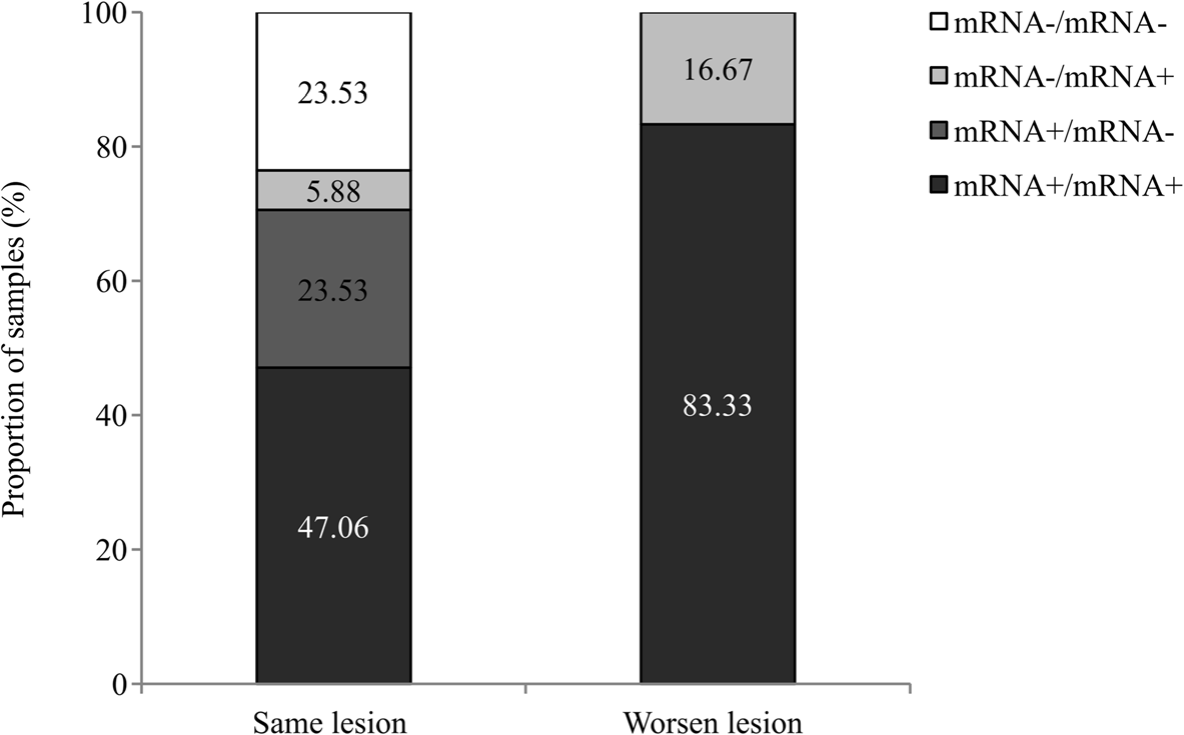
E6/E7 mRNA detection follow up in accordance with lesion progression in women that were tested twice. These women were tested twice (in 2012/2013 and 2014). Women were classified according to E6/E7 mRNA detection in two samples: the two specimens taken in consequent years were positive (mRNA+/mRNA+), the first sample was positive and the second one negative (mRNA+/mRNA-), both samples were negative (mRNA-/mRNA-) and first specimen was negative and the last one positive (mRNA-/mRNA+). Lesion progression was divided into two groups: women that had the same lesion during the time (same lesion) and women whose pathology had worsened over the time (worsen lesion)

Women whose diagnosis had worsened over time were mainly positive for mRNA in two analyzed samples (83.33 %) or had a previous negative result in mRNA but then mRNA was detected (16.67 %) (Fig. 4). Otherwise, in women whose pathological results did not vary over time, E6/E7 mRNA was not detectable in both samples in 23.53 % or mRNA was detected in the sample taken first but not in the second specimen in 23.53 %. Even so, mRNA in both samples was positive in 47.06 % of women with the same grade of lesion.

### Type of infection vs. oncogenes expression

Single HPV infection was detected in 40 of 105 analyzed women and 65 women were infected with multiple HPV infection. Among women with single HPV infection E6/E7 mRNA positivity rate was 67.50 % whereas that rate was 70.77 % in women with multiple HPV infection. The 60 % of women with single HPV infection had normal cytology results whereas the 40 % showed some lesion (high grade or low grade lesion). On the other hand, the 58.33 % of women with multiple HPV infection had some lesion and only the 41.66 % has normal cytology results. Among women with multiple HPV infection and positive for E6/E7 mRNA the 19.56 % were expressing more than one genotype oncogenes.

## Discussion

Human papillomavirus is closely related with cervical cancer. HPV infections usually show a regression in the next 2 years, but few patients infected with HR-HPV genotypes are at risk of developing cervical cancer [21, 22]. During carcinogenesis process, E6 and E7 oncoprotein overexpression is the necessary factor to develop cervical neoplasia [12]. To date, cervical cancer screening has been one of the most effective tools to prevent this disease and nowadays, DNA testing is growing worldwide. Nevertheless, DNA only gives information about virus presence but makes no difference between latent and active or persistent infections [17]. In persistent infections, E6 and E7 genes expression is not regulated and oncoproteins overexpression stimulates cancerous lesions development. Thus, it is extremely relevant to find cervical cancer prognosis biomarker to 1) anticipate the emergence of high-grade lesion, 2) improve screening programs and 3) decrease the amount of women referred to colposcopy needlessly.

DNA positivity rate was less valuable in clinical use than mRNA as it has been observed in previous studies [19, 23, 24]. HPV mRNA test is more specific but has less sensitivity than DNA test, hence, mRNA as triage test could reduce HPV DNA positive women sent to colposcopy in comparison with cytology although a lower sensitivity is assumed [19, 24]. Moreover, it has been observed that mRNA test plus cytology has more clinical relevance than DNA screening [23, 25].

Among mRNA positive women HPV 16 single infection transcript was the most recurrent, though, the main majority of women tested had multiple HPV infection. HPV 16 tended to express more than other genotypes, which may be connected with the fact that it is considered the most carcinogenic genotype [5]. Nevertheless, HPV-16 transcript could be the most recurrent owing to its high incidence in the samples (68.71 %). Moreover, after studying the expression of each genotype, HPV-33 was the most expressed genotype (100 %) followed by 18 and 16 (77.77 and 70.29 %, respectively). These results may be due to the low number of samples with HPV-33 but even so it should be take into account. Moreover, although the E6/E7 mRNA positivity rate was similar in women infected with single and multiple HPV infection it was observed a higher proportion of women with low or high grade lesion among women with multiple HPV infection. This result suggested that although oncogenes are expressing at the same percentage in both infection types, those women with multiple HPV infection have more probabilities to develop cervical lesion, it may be due to the interaction of different genotypes.

It was proved that E6/E7 mRNA positivity rate was higher in women with high-grade lesion than in samples with low-grade lesion or without lesion [8, 19, 26]. Besides, E6/E7 mRNA negativity rate was higher in women without lesion which means the possibility of an episomal state of the virus and effective regulation of transcription, which makes more likely spontaneous clearance of the infection. Moreover, as it was assessed in previous works, in ASCUS and LSIL samples, sensitivity is higher in HPV DNA test than in HPV mRNA test, but specificity is higher in mRNA test than DNA test. The same happens in samples with HSIL [19]. HPV mRNA test would gain specificity. Even so, this specificity gain could be related with the fact that amplification of viral mRNA is limited to few genotypes [27]. Moreover, these differences between specificity and sensitivity manifest the need for further works since, only a HPV test with high levels of sensitivity and specificity should be implemented for use in population screening programs [28].

According to E6/E7 mRNA as cervical disease biomarker, mRNA positivity increased over time in specimens with lesions in women tested once and in tested twice, namely, E6/E7 mRNA positive samples showed a malignancy progress during the next years. These results were expected since E6 and E7 oncogene are over expressed in precancerous lesions and it has been proved how these oncogenes and cervical lesion severity increases at the same time [29]. Therefore, E6/E7 mRNA may be a very useful biomarker for cervical disease considering that women who are E6/E7 mRNA positive should be monitored more closely although their cytological results were normal. Besides, women who only were tested once, in samples mRNA negative, it was observed an increase of women with ASCUS or LSIL during the next years. It may be because these samples were negative for mRNA when the samples was taken but over the time there was a viral transcription deregulation developing cervical lesions.

Nevertheless, in women who were tested twice, there was no difference in mRNA negativity between samples tested first and specimens tested one or two years later, so it seemed that there were a significant number of specimens that had minor cervical lesions but were negative for E6/E7 mRNA. This event could be related with the fact that low-grade cervical lesions normally disappears in a few months without treatment [30]. Even so mRNA test has been found more specific and clinically more useful than DNA test in women with low grade lesions [24].

Finally, E6/E7 mRNA could be a good prognosis marker since it was observed that women who were positive for E6/E7 mRNA had more probabilities to develop lesion progression. Besides, women in which samples mRNA was negative had more possibilities to remain the same pathological grade or have infection clearance. These findings are in concordance with previous studies where E6/E7 mRNA was considered a short-term prognostic factor for high-grade lesions [31]. Nevertheless, according to Discacciati et al., E6/E7 mRNA is not a long-term prognostic factor since women who were positive for DNA and negative for E6/E7 mRNA may become DNA and E6/E7 mRNA positive women which would change their pathological results [31].

## Conclusions

E6 and E7 oncogene mRNA detection seems to be a useful instrument as a triage test in women infected with HPV-16, -18, -31, -33 or -45 genotypes. Moreover, it could be a promising biomarker since it has been proved its relationship with lesion grade, making possible the reduction of HPV positive women referred to colposcopy. Even so, further studies are needed to use NucliSens® EasyQ® HPV v1 Test as standard screening method in HPV positive women.

## References

[1] Munoz N (2000). Human papillomavirus and cancer: the epidemiological evidence. J Clin Virol.

[2] Bray F, Ren J, Masuyer E, Ferlay J (2013). Global estimates of cancer prevalence for 27 sites in the adult population in 2008. Int J Cancer.

[3] Soerjomataram I, Lortet-Tieulent J, Parkin DM, Ferlay J, Mathers C, Forman D (2012). Global burden of cancer in 2008: a systematic analysis of disability-adjusted life-years in 12 world regions. Lancet.

[4] de Sanjose S, Quint WGV, Alemany L, Geraets DT, Ellen Klaustermeier J, Lloveras B (2010). Human papillomavirus genotype attribution in invasive cervical cancer: a retrospective cross-sectional worldwide study. Lancet Oncol.

[5] De Vuyst H, Clifford G, Li N, Franceschi S (2009). HPV infection in Europe. Eur J Cancer.

[6] Castellsagué X, Iftner T, Roura E, Vidart JA, Kjaer SK, Bosch FX (2012). Prevalence and genotype distribution of human papillomavirus infection of the cervix in Spain: The CLEOPATRE study. J Med Virol.

[7] Delgado D, Manuel Marin J, de Diego J, Guerra S, Gonzalez B, Barrios JL (2012). Human papillomavirus (HPV) genotype distribution in women with abnormal cervical cytology in the Basque Country, Spain. Enferm Infecc Microbiol Clin.

[8] Sjoeborg KD, Trope A, Lie AK, Jonassen CM, Steinbakk M, Hansen M (2010). HPV genotype distribution according to severity of cervical neoplasia. Gynecol Oncol.

[9] Wentzensen N, Schiffman M, Dunn T, Zuna RE, Gold MA, Allen RA (2009). Multiple human papillomavirus genotype infections in cervical cancer progression in the study to understand cervical cancer early endpoints and determinants. Int J Cancer.

[10] Carter J, Koutsky L, Hughes J, Lee S, Kuypers J, Kiviat N (2000). Comparison of human papillomavirus types 16, 18, and 6 capsid antibody responses following incident infection. J Infect Dis.

[11] Frazer IH (2009). Interaction of human papillomaviruses with the host immune system: a well evolved relationship. Virology.

[12] Wechsler EI, Wang Q, Roberts I, Pagliarulo E, Jackson D, Untersperger C (2012). Reconstruction of human papillomavirus type 16-mediated early-stage neoplasia implicates E6/E7 deregulation and the loss of contact inhibition in neoplastic progression. J Virol.

[13] von Knebel Doeberitz M, Rittmuller C, Aengeneyndt F, Jansen-Durr P, Spitkovsky D (1994). Reversible repression of papillomavirus oncogene expression in cervical-carcinoma cells - consequences for the phenotype and E6-P53 and E7-Prb interactions. J Virol.

[14] Vaccarella S, Lortet-Tieulent J, Plummer M, Franceschi S, Bray F (2013). Worldwide trends in cervical cancer incidence: impact of screening against changes in disease risk factors. Eur J Cancer.

[15] Bray F, Loos A, McCarron P, Weiderpass E, Arbyn M, Moller H (2005). Trends in cervical squamous cell carcinoma incidence in 13 European countries: changing risk and the effects of screening. Cancer Epidemiol Biomarkers Prev.

[16] Cuzick J, Clavel C, Petry K, Meijer CJLM, Hoyer H, Ratnam S (2006). Overview of the European and North American studies on HPV testing in primary cervical cancer screening. Int J Cancer.

[17] Wright TC, Massad S, Dunton CJ, Spitzer M, Wilkinson EJ, Solomon D (2007). 2006 Consensus guidelines for the management of women with abnormal cervical cancer screening tests. Obstet Gynecol.

[18] Ho CM, Lee BH, Chang SF, Chien TY, Huang SH, Yan CC (2010). Type-specific human papillomavirus oncogene mRNA levels correlate with the severity of cervical neoplasia. Int J Cancer.

[19] Benevolo M, Vocaturo A, Caraceni D, French D, Rosini S, Zappacosta R (2011). Sensitivity, specificity, and clinical value of human papillomavirus (HPV) E6/E7 mRNA assay as a triage test for cervical cytology and HPV DNA test. J Clin Microbiol.

[20] Szarewski A, Mesher D, Cadman L, Austin J, Ashdown-Barr L, Ho L (2012). Comparison of seven tests for high-grade cervical intraepithelial neoplasia in women with abnormal smears: the Predictors 2 study. J Clin Microbiol.

[21] Evander M, Edlund K, Gustafsson A, Jonsson M, Karlsson R, Rylander E (1995). Human papillomavirus infection is transient in young women: a population-based cohort study. J Infect Dis.

[22] Schiffman M, Herrero R, Desalle R, Hildesheim A, Wacholder S, Rodriguez AC (2005). The carcinogenicity of human papillomavirus types reflects viral evolution. Virology.

[23] Tezcan S, Ozgur D, Ulger M, Aslan G, Gurses I, Serin MS (2014). Human papillomavirus genotype distribution and E6/E7 oncogene expression in turkish women with cervical cytological findings. Asian Pac J Cancer Prev.

[24] Sørbye SW, Fismen S, Gutteberg TJ, Mortensen ES, Skjeldestad FE (2014). HPV mRNA is more specific than HPV DNA in triage of women with minor cervical lesions. PLoS One.

[25] Spathis A, Kottaridi C, Chranioti A, Meristoudis C, Chrelias C, Panayiotides IG (2012). mRNA and DNA detection of human papillomaviruses in women of all ages attending two colposcopy clinics. PLoS One.

[26] Perez Castro S, Iñarrea Fernández A, Lamas González MJ, Sarán Diez MT, Cid Lama A, Alvarez Martín MJ (2013). Human papillomavirus (HPV) E6/E7 mRNA as a triage test after detection of HPV 16 and HPV 18 DNA. J Med Virol.

[27] Boulet GA, Micalessi IM, Horvath CA, Benoy IH, Depuydt CE, Bogers JJ (2010). Nucleic acid sequence-based amplification assay for human papillomavirus mRNA detection and typing: evidence for DNA amplification. J Clin Microbiol.

[28] Ovestad IT, Vennestrøm U, Andersen L, Gudlaugsson E, Munk AC, Malpica A (2011). Comparison of different commercial methods for HPV detection in follow-up cytology after ASCUS/LSIL, prediction of CIN 2–3 in follow up biopsies and spontaneous regression of CIN2–3. Gynecol Oncol.

[29] Benevolo M, Terrenato I, Mottolese M, Marandino F, Carosi M, Rollo F (2011). Diagnostic and prognostic validity of the human papillomavirus E6/E7 mRNA test in cervical cytological samples of HC2-positive patients. Cancer Causes Control.

[30] Crow JM (2012). HPV: the global burden. Nature.

[31] Discacciati MG, da Silva ID, Villa LL, Reis L, Hayashi P, Costa MC (2014). Prognostic value of DNA and mRNA E6/E7 of human papillomavirus in the evolution of cervical intraepithelial neoplasia grade 2. Biomark Insights.

